# Autoimmune Calcium Channelopathies and Cardiac Electrical Abnormalities

**DOI:** 10.3389/fcvm.2019.00054

**Published:** 2019-05-02

**Authors:** Yongxia Sarah Qu, Pietro Enea Lazzerini, Pier Leopoldo Capecchi, Franco Laghi-Pasini, Nabil El Sherif, Mohamed Boutjdir

**Affiliations:** ^1^Department of Cardiology, New York Presbyterian Brooklyn Methodist Hospital, Brooklyn, NY, United States; ^2^VA New York Harbor Healthcare System and State University of New York Downstate Medical Center, Brooklyn, NY, United States; ^3^Department of Medical Sciences, Surgery and Neurosciences, University of Siena, Siena, Italy; ^4^NYU School of Medicine, New York, NY, United States

**Keywords:** calcium channel, autoantibodies, autoimmune, channelopathy, cardiac electrical abnormalities

## Abstract

Patients with autoimmune diseases are at increased risk for developing cardiovascular diseases, and abnormal electrocardiographic findings are common. Voltage-gated calcium channels play a major role in the cardiovascular system and regulate cardiac excitability and contractility. Particularly, by virtue of their localization and expression in the heart, calcium channels modulate pace making at the sinus node, conduction at the atrioventricular node and cardiac repolarization in the working myocardium. Consequently, emerging evidence suggests that calcium channels are targets to autoantibodies in autoimmune diseases. Autoimmune-associated cardiac calcium channelopathies have been recognized in both sinus node dysfunction atrioventricular block in patients positive for anti-Ro/La antibodies, and ventricular arrhythmias in patients with dilated cardiomyopathy. In this review, we discuss mechanisms of autoimmune-associated calcium channelopathies and their relationship with the development of cardiac electrical abnormalities.

## Introduction

Voltage gated calcium channels (VGCCs) are macromolecular complexes which include the main pore forming α_1_-subunits, the accessory β, α_2_δ, and γ-subunits (1–4). In the heart, VGCCs mediate calcium (Ca) influx in response to membrane depolarization and modulate excitability, contraction, hormonal secretion and gene transcription (1–6). There are many pathologies, both genetic and acquired, involving VGCCs. Mutations in VGCCs cause dysfunctions of Ca channels, resulting in abnormal excitation of the cardiomyocyte, and cardiac arrhythmias (2, 6–8), which contribute substantially to morbidity and mortality. Among the different pathophysiological mechanisms of arrhythmogenesis, a new area of interest has recently emerged and is related to autoimmune-associated Ca channel dysfunction (autoimmune Ca channelopathies) in cardiac arrhythmias (9–12). This review summarizes the recent findings on the roles of cardiac Ca channels in autoantibodies-associated cardiac arrhythmias.

## Voltage-Gated Calcium Channels in the Heart

L-type and T-type Ca channels are the two major classes of VGCCs in the heart. The L-type Ca channel is a high voltage-activated, long-lasting, and the T-type channel is characterized by a low voltage-activated, transient-type channel (2, 3, 5, 6, 13, 14). There are 10 isoforms of mammalian genes encoding the α_1_ subunit. (5, 15–18). *CACNA1S, CACNA1C, CACNA1D*, and *CACNA1F* encode α_1S_, α_1C_, α_1D_, and α_1F_ subunits (L-type Ca channels) respectively. *CACNA1A, CACNA1B*, and *CACNA1E* encode α_1A_, α_1B_, and α_1E_ subunits (P/Q-, N-, and R-types), respectively, (19–21). The T-type Ca channels α_1G_, α_1H_, and α_1I_ subunits are encoded by *CACNA1G, CACNA1H*, and *CACNA1I*, respectively, (22–24). Among these channels, the L-type Ca channels α_1C_ and α_1D_ isoforms and the T-type Ca channels α_1G_ and α_1H_ isoforms are the major VGCCs expressed in the heart (25–27). The features and tissue distribution of the L-type and T-type Ca channels are summarized in Table 1.

**Table 1:** Features of Ca channels in the heart.

**Channel**	**Gene**	**Activation**	**Distribution**	**Developmental change**	**Function**
α_1C_ VGCC	Cav1.2	−40 mV	Ubiquitous	Increase with developmental stage	• Action potential in SAN and AVN, • Inotropy, contraction of atria and ventricles
α_1D_ VGCC	Cav1.3	−60 mV	SAN, AVN, Atria in adult heart; Ubiquitous in immature heart	Decrease with developmental stage	• Pace making, • AVN conduction • Atrial excitability
α_1G_ VGCC	Cav3.1	−70 mV	Supraventricular tissue, 30-fold more in SAN than in atria	Increase during development, maximal at adult stage	• Pacing making • AVN conduction
α_1H_ VGCC	Cav3.2	−70 mV	Supraventricular tissue	Predominant in embryonic stage	

*references (6, 14, 15, 17, 18, 28–35)*.

### L-type Ca Channels in the Heart

#### α_1C_ L-type Ca Channel

Cardiac α_1C_ L-type VGCC is a protein complex comprised of α_1C_, β_2_, and α_2_/δ subunits. The α_1_ subunit is the pore-forming subunit, which determines the major features of the channel, such as ion selectivity, activation-inactivation and the sensitivity to Ca channel blockers (3, 6, 15, 16). The β_2_ and α_2_/δ accessory subunits play important roles in the regulation of the biophysical properties of Ca channels (36). The α_1C_ VGCC is universally expressed in the heart and plays a critical role in excitation–contraction coupling, impulse generation in sinus node (SAN) and its conduction in the atrioventricular node (AVN). The Ca ions entering the cardiomyocytes through α_1C_ VGCCs also shape the plateau phase of the ventricular action potential and induce the release of Ca from the sarcoplasmic reticulum (calcium induced-calcium release) which initiates the myocardial contraction (1, 6, 36).

#### α_1D_ L-type Ca Channel

In contrast to the ubiquitously expressed α_1C_ VGCCs in the heart, α_1D_ VGCCs are restricted to the supraventricular tissue of the adult heart, with the highest expression in the atria, SAN, and AVN, but they are not expressed in the normal adult ventricles (5, 28, 37–42). In the fetal heart, however, α_1D_ VGCCs are expressed throughout the heart including the ventricles, atria, SAN, and AVN (39). While α_1C_ VGCCs activate at more positive (−40 and −30 mV) potentials, α_1D_ VGCCs activate between −60 and −40 mV at a range of diastolic depolarization of the SAN (28, 42). This unique feature allows α_1D_ VGCCs to play an important role in the automaticity of SAN pacemaker cells (29, 43, 44). The unexpected SAN dysfunction reported in mice lacking α_1D_ VGCCs was the first evidence of their importance in heart automaticity (28, 42, 44). Deletion of the α_1D_ VGCC gene impairs pace making in the SAN and atrioventricular conduction in the AVN but has no effect on myocardial contractility (42, 44).

### T-type Ca Channels in the Heart

There are 3 isoforms of T-type VGCC: α_1G_ (23, 45), α_1H_ (24), and α_1I_ (45, 46). Among them, α_1G_ and α_1H_ are the major isoforms in the myocardium and their expression is developmentally regulated (17, 30, 31). While α_1H_ T-type VGCC constitutes the predominant isoform in embryonic heart tissue (32); α_1G_ T-type VGCC expression increases during the perinatal period and reaches its maximal level in adulthood. In adult SAN, α_1G_ expression is higher than α_1H_ T-type VGCC (26, 27, 33). In contrast to α_1D_ L-type VGCC, which requires accessary subunits for normal gating, α_1G_ or α_1H_ subunits expression alone exhibit native T-type Ca channel properties (17, 47, 48). In addition, T-type VGCCs open at significantly more negative membrane potentials that overlap the pacemaker potentials of SAN cells (30, 49). The threshold for activation is −70 to −60 mV, and the channel is fully activated at −30 to −10 mV (17, 31, 49). T-type VGCCs are expressed in the SAN (34), the AVN (50), and the Purkinje fibers (51, 52), supporting their roles in the generation of the diastolic depolarization, the automaticity of SAN and the impulse conduction of the heart (30, 31, 53, 54). Indeed, homozygous transgenic mice lacking α_1G_ VGCC exhibit first-degree atrioventricular block (AVB) and bradycardia (25). Collectively, both L-type, and T-type Ca channels by virtue of their tissue-specific localization can modulate automaticity, conduction and repolarization, and as such, agents and compounds like autoantibodies (discussed below) which interact and target these channels are expected to affect the electrical activity of the heart.

## Autoantibodies-Associated Cardiac Calcium Channelopathies

Autoimmune disorders and cardiovascular disorders are associated with significant morbidity and mortality and are a major health problem both in the USA and worldwide. While the field of “cardio-immunology” is being formally established, recent and emerging advances in this area indicate that autoantibodies play an important role in the development of cardiac arrhythmias.

### Autoantibodies Against Ca Channel and Ventricular Arrhythmias: Anti-α_1C_ Subunit Antibody

Autoimmunity is one of the main mechanisms involved in the pathogenesis of dilated cardiomyopathy (DCM) (55–57). Sudden death caused by ventricular arrhythmias is one of the leading causes of death in patients with DCM (58–60). Results from previous studies indicated that the VGCC plays an important role in the pathogenesis of DCM (11, 61, 62). The function of VGCCs in DCM is affected either by autoantibodies directed against the regulatory pathway/accessary subunits or autoantibodies targeting the pore forming α_1_ subunit itself. Several autoantibodies indirectly affecting the L-type VGCCs have been identified in patients with DCM (63–65). The presence of antibody against the β-adrenoceptor was first reported in a patient with Chagas' disease by Sterin-Borda et al. (66). Ten years later, Wallukat and Wollenberger demonstrated the presence of an agonist-like anti-β1 adrenoceptor in DCM patients (67). Subsequent studies showed that these autoantibodies in DCM target the second extra-cellular loop of the β1-adrenoreptor (68), resulting in a positive chronotropic effect. Autoantibodies against β1-adrenoceptors were closely related to ventricular arrhythmias in patients with DCM (69). Anti-β1-adrenoceptor antibodies induced in an animal model caused action potential duration prolongation, with higher propensity for induction of early repolarization, promoting the development of ventricular arrhythmias which increased the risk of sudden death (69–71). Notably, Christ et al. (72) demonstrated that anti-β1 adrenoceptor antibodies increased L-Type Ca current, I_Ca−L_ in adult rat ventricular cells in concordance with the prolongation of the action potential duration. Autoantibodies against adenine nucleotide translocators, which cross-react with VGCCs, increases the Ca inflow which causes myocyte damage by Ca overload in DCM (73–75).

**Figure 1:**
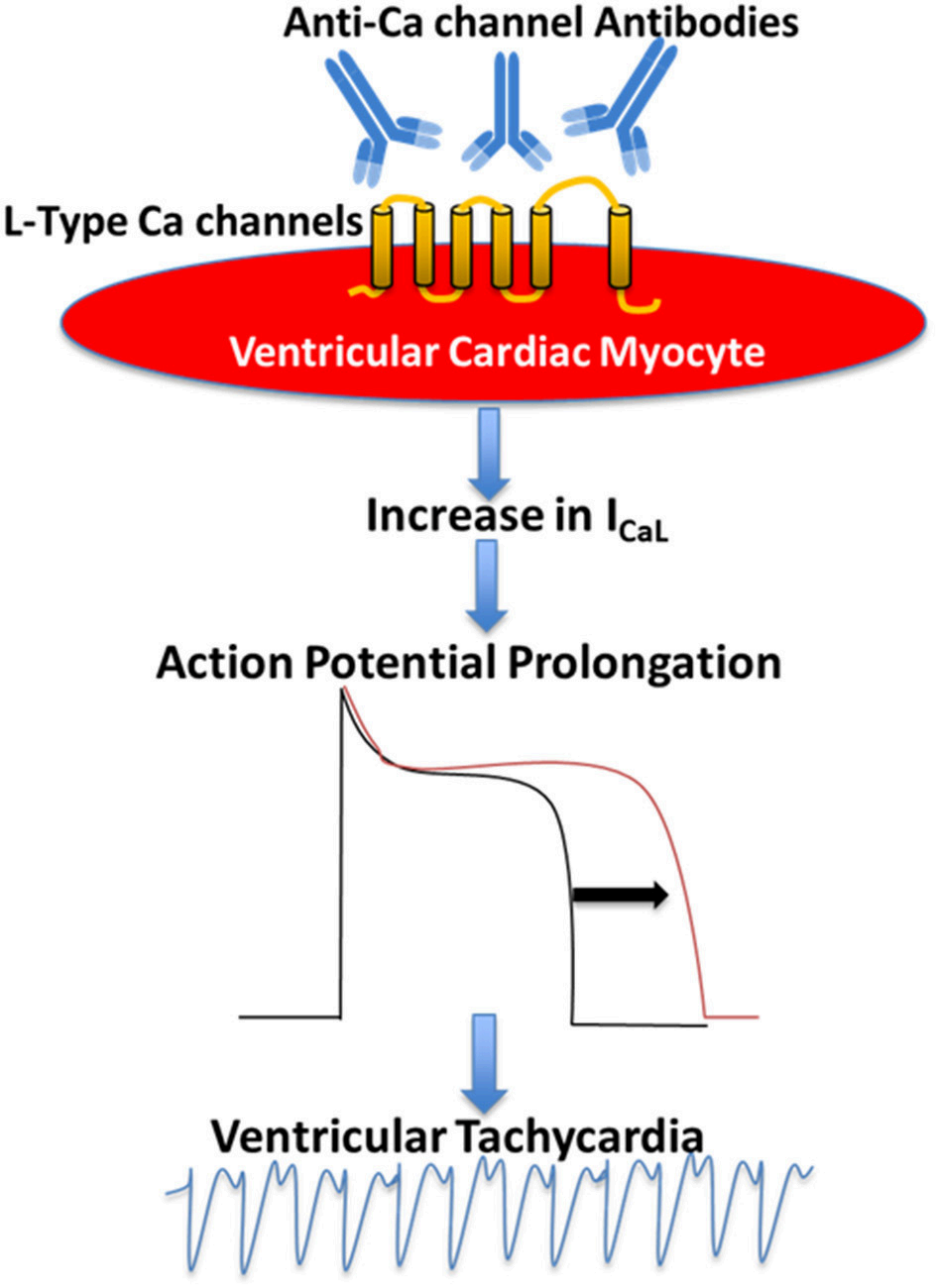
Proposed mechanism of the pathogenic role of anti-Ca channels autoantibodies in Dilated Cardiomyopathy. Anti-Ca channels autoantibodies target L-type Ca channels in the ventricular myocyte resulting in an increase in L-type Ca current (I_CaL_) which in turn leads to action potential prolongation and ventricular arrhythmias.

The evidence of the presence of agonist-like autoantibodies directly against the L-type VGCC α_1C_ subunits in DCM was demonstrated by Liao et al. (76) and Xiao et al. (11) subsequently demonstrated that autoantibodies against α_1C_ Ca channel are arrhythmogenic and lead to sudden cardiac death in patients with DCM. In a prospective study, the authors compared ventricular arrhythmias and sudden death in 80 patients with DCM and age- and gender-matched controls for 32 months. Autoantibodies against L-type α_1C_ subunits (anti-α_1C_) were detected by ELISA in 39 patients with DCM (48.8%) and 5 controls (6.3%). Higher incidence of ventricular arrhythmias and sudden cardiac death was observed in anti-α_1C_ antibody-positive patients as compared to the antibody-negative patients. The presence of anti-α_1C_ antibodies was identified as the strongest independent predictor for sudden death in DCM (11). The arrhythmogenic effect of anti-α_1C_ antibodies was reproduced in a rat model (11). Perfusion of affinity purified anti-α_1C_ antibodies lead to ventricular arrhythmias by action potential duration prolongation and triggered activity (11). This effect was blocked by pre-incubating the anti-α_1C_ antibodies with its specific peptide and Ca channel blockers, indicating the specificity of the arrhythmogenic effect of the anti-α_1C_ antibodies (11). To further investigate the underlying mechanism of the anti-α_1C_ antibodies, Xiao et al. using immunofluorescent approach demonstrated that anti-α_1C_ antibodies were able specifically to bind to the Ca channel on the myocyte, enhancing the channel's activities (hence the agonist-like effect). In a prospective study, Yu et al. (62) recruited 2096 patients with congestive heart failure, of which 841 dilated cardiomyopathy patients (DMC) 1,255 ischemic cardiomyopathy (ICM) patients, and 834 controls. By the end of a median follow up of 52 months, 102 cases of DCM had sudden cardiac death. Interestingly, the rate of anti-Ca channel antibody in DCM was significantly higher in DCM patients compared to controls. After adjusting for risk factor including age, left ventricular ejection fraction (LVEF), hypertension, diabetes, New York Heart Association (NYHA) functional classification, QTc, and medications, Cox regression analysis revealed that the presence of anti-Ca channel antibodies still remains an independent risk factor for sudden cardiac death in DCM patients. In conclusion, there are novel agonist-like anti-α_1C_ Ca channel antibodies in patients with DCM, which prolong action potential duration and QT interval, induce early after depolarizations, and ventricular tachycardia, eventually leading to sudden cardiac death. These antibodies could serve as novel clinical markers and as positive predictor of sudden death in DCM (Figure 1) (61, 62).

### Autoimmune-Associated Brady-Arrhythmias and Conduction Abnormalities: Cardiac L-type Ca Channels and Anti-ro Antibodies

While presence of the anti-α_1C_ Ca channel antibody is identified as a strong predictor for ventricular arrhythmias and sudden cardiac death in DCM (11), its role has not been well-established in other autoimmune-associated cardiac electrical abnormalities. The best studied disease caused by autoantibody related L-type Ca channel dysfunction is autoimmune-associated congenital heart block (CHB) characterized by AVB, and sinus bradycardia (10, 35, 77–80). CHB is a conduction abnormality that affects structurally normal hearts of fetuses and/or newborn to mothers with autoantibodies against the intracellular ribonucleoproteins SSA-Ro and SSB-La (10, 79, 80). The hallmark of CHB is various degrees of AVB, with complete AVB being the most common, for which more than 60% of affected children require lifelong pacemakers (81), and carries mortality rate up to 30% (81, 82). Because anti-Ro antibodies are the most prevalent autoantibodies in CHB (83–85), anti-La antibodies are not discussed in this review. There are 2 subtypes of anti-Ro autoantibodies: anti-52 and anti-60 kD SSA/Ro (collectively termed anti-Ro antibodies in this review). Anti-Ro antibodies result from an autoimmune response to the SSA-Ro antigen, which is an intracellular ribonucleoprotein that is not accessible to the circulating anti-Ro antibodies in the normal cardiac myocyte, likely because of their large size. Anti-Ro antibodies are more prevalent in certain autoimmune diseases including Sjögren's syndrome, systemic lupus erythematosus, scleroderma, rheumatoid arthritis, systemic sclerosis, and myositis (86, 87). Intriguingly, these anti-Ro antibodies are also present in the general healthy population (87–89). The incidence of CHB is about 1:11,000 (81, 90); however, this incidence dramatically increases to about 5% in anti-Ro positive mothers and up to 18% in subsequent pregnancies thereby affecting the decision to have a second child (79, 81). The causal relationship of anti-Ro antibodies to the development of CHB was reproduced in both active and passive mice models of CHB (81, 91–93). Various degree of AVB developed in pups born to female mice immunized with recombinant 52 SSA/Ro protein (active immunization) (81, 93, 94). Transfer of anti-Ro antibodies from mothers with CHB children (anti-Ro antibody positive IgG) directly into timely pregnant mice also resulted in first degree AVB and, surprisingly, sinus bradycardia in about 70% of the pups (passive immunization) (91). Similarly, clinical data (95, 96) also confirmed similar sinus bradycardia in newborns of mothers with anti-Ro antibody positive IgG, indicating that the spectrum of CHB extends beyond AVN to also affect SAN.

#### Anti-Ro Antibody Positive IgG Inhibits Both α_1C_ and α_1D_ Ca Currents

As mentioned above, the hallmark of CHB is AVB. The conduction of the impulse through the AVN depends critically on α_1C_ Ca current, I_Ca−L_, which activates at more positive (−40 and −30 mV) potentials (97). It is logical to speculate that anti-Ro antibody positive IgG might target α_1C_ Ca channel to disturb the electrical conduction at AVN as seen in CHB. Anti-Ro antibody positive IgG and affinity purified anti-52 Ro antibodies from mothers with CHB children, but not anti-Ro antibody negative IgG from healthy mothers, inhibited I_Ca−L_ in isolated SAN, AVN cells, Purkinje fibers and in ventricular cells by 50–59% (77, 78, 98–100). In addition, anti-Ro antibody positive IgG had no effect on K currents (the transient outward current, I_to_ and the inward rectifier, I_K1_), or the Na current (I_Na_), indicating its specificity toward Ca channels (98). To exclude the possibility of potential contamination from other ion currents, α_1C_ Ca channels expressed in Xenopus oocytes were similarly inhibited about 50% by anti-Ro antibody positive IgG (92, 99, 100).

While inhibition of α_1C_ I_Ca−L_ could account for the AVB seen in CHB, the contribution of α_1C_ I_Ca−L_ to diastolic depolarization of the SA node is generally considered to be minor. SAN pacemaker depolarization occurs between −60 and −40 mV; however α_1C_ I_Ca−L_ activates at more positive (−40 and −30 mV) potentials (101). Knockout of the α_1D_ Ca channel, which activates at −60 and −40 mV in mice, results in significant sinus bradycardia and AVB (28, 42, 102), a phenotype reminiscent to that seen in CHB. Mangoni et al. (44) showed I_Ca−L_ in SAN cells was decreased by 75% in α_1D_ Ca channel knockout mice compared with wild-type mice, which indicates that the contribution of the α_1D_ Ca channel to total I_Ca−L_ is significant in the mouse SA node cell. Furthermore, our previous studies demonstrated that both α_1D_ Ca channel transcripts and proteins are expressed in human fetal heart and in adult rabbit SAN (39, 40). Collectively, these data suggest that α_1D_, along with α_1C_, contribute to form I_Ca−L_, playing a critical role in pace making activity in SAN and are a potential target by anti-Ro antibodies. Because there are no biophysical methods or specific blockers to separate α_1D_ from α_1C_ I_CaL_ in native cells, the specific effect of anti-Ro antibodies on α_1D_ I_Ca−L_ has been challenging. Initial studies were carried out in expression systems to allow individual expression of α_1D_ I_Ca−L_ to characterize the effect of anti-Ro antibody positive IgG_._ Anti-Ro antibody positive IgG from mothers with CHB children inhibited α_1D_ I_Ca−L_ by about 43% in tsA201 cells and about 33% in Xenopus oocytes (40, 77, 78, 92, 99, 100). To overcome this limitation of using expression systems, our group has tested the effect of anti-Ro antibodies on α_1D_ I_Ca−L_ in native neonatal cardiomyocytes, in which the α_1C_ gene was effectively silenced by lentivirus. Adding anti-Ro antibody positive IgG resulted in 35% reduction of α_1D_ I_Ca−L_ in naïve cardiomyocytes (103), similar to the results seen using expression systems.

**Figure 2:**
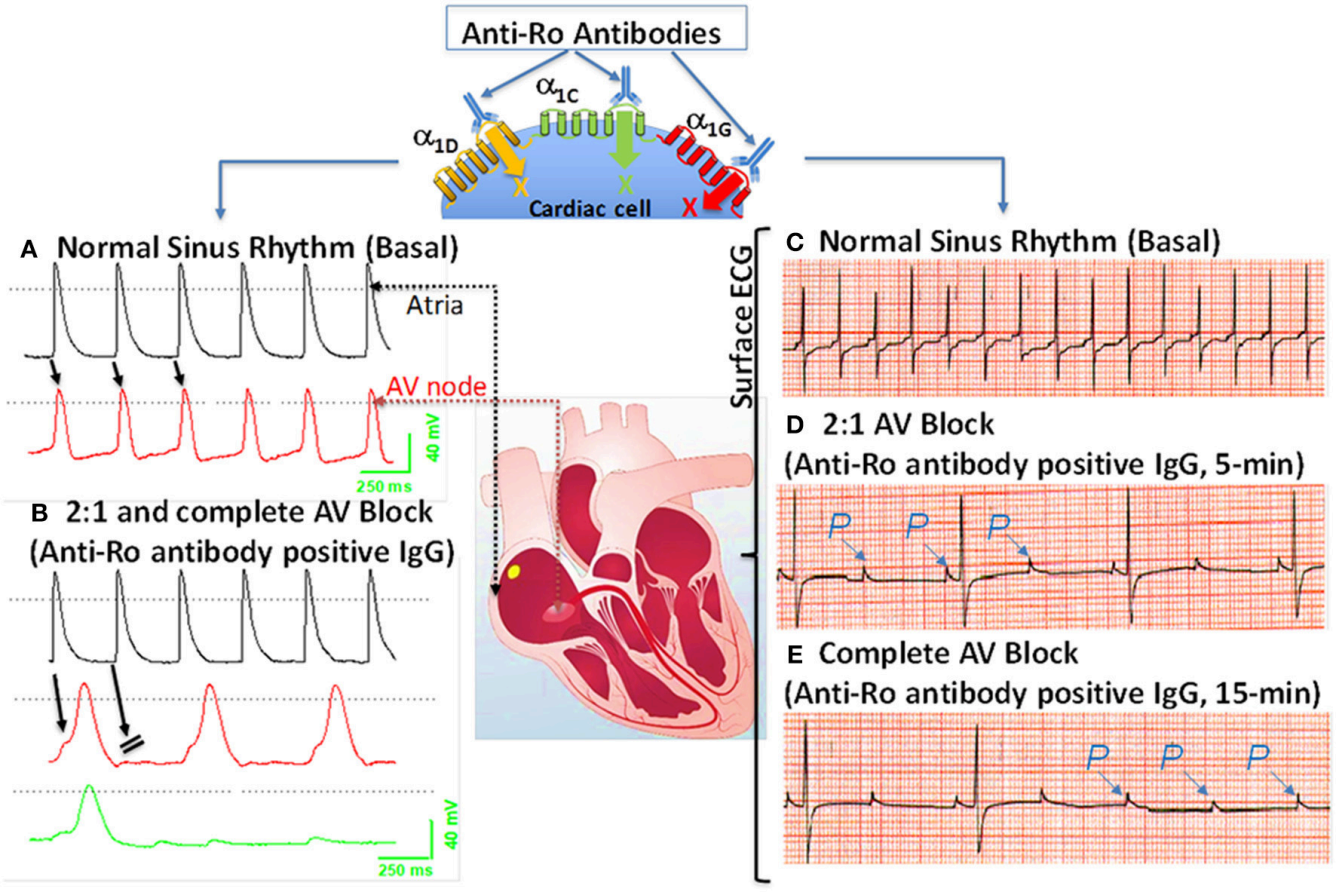
Effects of anti-Ro antibodies from mothers of children with congenital heart block on an isolated multicellular AV nodal preparation (left) and Langendorff perfused whole heart (Right). **(A)** Simultaneous control action potentials from the crista terminalis (black tracing) and the AV node area (red tracing). **(B)** Superfusion of the preparation with positive IgG (800 μg/mL) for 10 min resulted in 2:1 AV block (indicated by the arrows) which progressed to near complete inhibition of the AV node action potential by 15 min **(B)**, (green tracing). **(C)** ECG was recorded by the conventional ECG machine in lead I, except for the use of silver wires at the recording end of the leads. One lead was inserted in the atrium, the second in the left ventricle near the apex, and the third in Tyrode's solution (ground). “P” indicates, the P wave and on the ECG. Regular sinus rhythm (horizontal scale, 50 mm/s and vertical scale: 5 mm/mV) at 300 beats/min in Tyrode's solution. **(D)** After 5 min of perfusion with positive IgG (800 μg/mL), there was bradycardia associated with a 2:1 second degree AV block that degenerated into complete AV block by 15 min of IgG perfusion **(E)**. The sectioned heart in the middle panel illustrates the location of the microelectrode recordings.

Because anti-Ro antibodies inhibit both α_1C_ and α_1D_ I_Ca−L_, it is anticipated that anti-Ro antibodies will cause both sinus bradycardia and AVB. Further experimental evidence using isolated multicellular AVN preparations (Figures 2A,B) and Langendorff-perfused whole hearts (Figures 2C–E) demonstrated that anti-Ro antibody positive IgG resulted in bradycardia associated with 2:1 AVB then complete third degree AVB as recorded by surface EG. In contrast, perfusion of the AVN preparation or whole heart with control anti-Ro antibody negative IgG had no effect on ECG parameters (78). The sinus bradycardia and AVB were also demonstrated in Langendorff-perfused human hearts by our group (77) and by others (104, 105). Similar findings were obtained using the optical mapping technique, which allows simultaneous recording of voltage action potentials at multiple areas of the heart including the AVN area. Perfusion of hearts with anti-Ro antibody positive IgG revealed the sites of conduction abnormalities at the sinoatrial junction and AVN, thereby confirming the site of action for these autoantibodies (106).

In summary, α_1D_ and α_1C_ Ca channels both contribute to total I_Ca−L_ in the heart, with α_1D_ Ca channels playing a more critical role in the SAN and α_1C_ Ca channels in the AVN. Anti-Ro antibodies inhibit I_Ca−L_ emanating from both α_1D_ and α_1C_, resulting in AVB and sinus bradycardia seen in CHB. This causal relationship was confirmed by reproducing active and passive mice CHB models by induction of anti-Ro antibodies (active immunization) or passive transfer of the anti-Ro positive maternal IgG into pregnant mice (passive immunization). Altogether, anti-Ro autoantibodies' inhibition of Ca channels are causally related to the development of CHB, but the low incidence of CHB children born to anti-Ro antibodies positive mothers suggest that additional factor(s) may be necessary to contribute to the full spectrum of CHB.

#### Anti-ro Antibody Positive IgG Inhibits Ca Currents by Binding Directly to the Pore Forming Subunit of the Ca Channels

As pointed out earlier, anti-Ro antibody positive IgG cannot cross the sarcolemma of a normal fetal cardiac myocyte, and hence one can suspect that its effects are not directly mediated through its antigen, SSA/Ro, but rather via sarcolemma targets such as Ca channels. Evidence for direct interaction between anti-Ro antibodies and Ca channels is provided by the direct binding of anti-Ro antibodies on the pore forming α_1_ subunit of VGCC, resulting in inhibition of I_CaL_. Indeed, using immunostaining and Western blots, it was demonstrated that anti-Ro antibody positive IgG binds directly to the Ca channels' α_1_ subunit (99, 107). In a subsequent study, purified GST fusion proteins corresponding to the extracellular loop S5–S6 of each of the four domains that form the pore of the α_1D_ subunit were expressed and their reactivity to anti-Ro antibody positive IgG was tested. Fourteen percent of anti-Ro antibody positive IgG reacted specifically with the extracellular loop S5-S6 of the first domains of the α_1D_ subunit, as demonstrated by both ELISA and Western blots (108). L-type Ca channels' inhibition by anti-Ro antibodies is one of the mechanisms for the electrocardiographic abnormalities seen in CHB. The resulting formulation of the “Ca channel hypothesis” was based on the above experimental findings and was driven by the fact that AVN electrogenesis depends on the L-type Ca channels. Inhibition of this channel will ultimately lead to AVB, as seen in CHB. The “Ca channel hypothesis” states that circulating maternal antibodies directly cross react with L-type Ca channel pore forming protein α_1_-subunit, inhibiting the currents and leading to the development of AVB (97).

### T-type Ca Channel and Autoimmune-Associated Congenital Heart Block

T-type α_1G_ VGCCs subtype participates with α_1H_ in regulating electrical conduction through the AVN (18, 27, 31, 34). α_1G_ VGCC is highly expressed in the AVN in human hearts (27, 31, 32). Homozygous α_1G_ knockout mice exhibit first-degree AVB and bradycardia, a phenotype seen in CHB (25). These findings suggest α_1G_ VGCC as an additional potential cross-reactive target with anti-Ro antibody positive IgG in the development of CHB. Hu et al. demonstrated that anti-Ro antibody positive IgG decreased both I_CaL_ and T-type Ca current (I_Ca−T_) without affecting the delayed rectifier K current, I_K_, and the funny current, I_f_, in rabbit SAN cells (98). The average inhibition of I_Ca−T_ by anti-Ro antibody positive IgG was 31.4% at −40 mV and 44.1% at −20 mV in rabbit SAN cells (98). In addition, although anti-Ro antibody positive IgG inhibited the α_1H_ I_CaT_ expressed in the Xenopus oocyte (100), α_1H_ Ca channel knockout mice have no ECG changes (109), likely secondary to the low level of α_1H_ expression in the human neonatal AVN cells (107). These findings support the conclusion that the α_1G_ Ca channel is the target for anti-Ro antibody positive IgG. Strindberg et al. demonstrated α_1G_ mRNA and proteins in human fetal hearts and that α_1G_ I_Ca−T_ rather than α_1H_ I_Ca−T_ is the dominant current in the AVN in newborns (107). Experimental data using immunoprecipitation, Western blot and immunofluorescent staining have demonstrated accessibility of anti-Ro antibody positive IgG to the α_1G_ epitope on the surfaces on the cardiomyocytes in the human fetal heart (107). Reactivity to α_1G_ T-type VGCC was significantly higher in CHB maternal sera compared to controls. Binding epitope of anti-Ro antibody positive IgG was mapped to the extracellular S5–S6 portion of repeat I of α_1G_ subunit (aa305–319; designated as p305). Using the patch-clamp technique, the authors also demonstrated that anti-Ro antibody positive IgG inhibited I_Ca−T_ in isolated mice SAN cells (107). Taken together, these results indicate that anti-Ro antibody positive IgG readily target an extracellular epitope of α_1G_ T-type VGCC and inhibit the current in human fetal cardiomyocytes, thus contributing to the development of AVB as seen in CHB.

Anti-52kD Ro antibodies are present in 80% of mothers of children with CHB; however, the risk of having CHB children is low, with only 1–2% in single anti-Ro antibody positive pregnancies (84). Markham et al. investigated if reactivity with p305 (anti-Ro/p305) can be used clinically to more accurately predict CHB in anti-Ro antibody positive patients (110). Using anti-Ro antibody positive IgG and with multiple control groups, reactivity was determined and compared for binding to anti-Ro/p305. In mothers carrying anti-Ro antibodies, positive anti-Ro/p305 antibodies were detected in 3/59 (5%) CHB pregnancies, 4/30 (13%) unaffected pregnancies with a CHB-sibling, and 0/42 (0%) of unaffected pregnancies with no CHB-sibling. Similarly, using umbilical blood from 61 CHB and 41 healthy with CHB-sibling, in which reactivity would unambiguously substantiate exposure to maternal antibody, no association of anti-Ro/p305 with CHB was detected. These data indicate that anti-Ro/p305 reactivity in pregnant anti-Ro antibody-positive patients is not a robust maternal marker for assessing increased risk of CHB (110).

As described above, it is well-recognized that maternal anti-Ro antibody is associated with the development of the congenial AVB, at least in part resulting from an inhibitory cross-reaction with L- and T-type Ca channels. More recent, studies demonstrated that 10–60% of anti-Ro-positive subjects are at increased risk of developing QTc prolongation as a result of anti-Ro antibodies' interference with K channels, (111–115) resulting in complex ventricular arrhythmia, (116, 117) including Torsade's de Pointes (TdP) (118, 119). Lazzerini et al. (119) recently evaluated 25 consecutive patients who experienced TdP, where anti-Ro antibody was present in 15 out of 25 patients. Purified anti-Ro positive IgG from TdP patients cross-reacted with the Human Ether-a-go-go-related Gene (hERG) K channel and significantly inhibited the resulting current, IKr. This observation indicates that anti-Ro antibodies may represent a novel, clinically silent risk factors for TdP. To date, studies on the association of anti-Ro antibodies and atrial fibrillation are scarce. In our previous study (120), we were able to induce atrial fibrillation in the α_1D_ knockout mice but not in the wild-type mice. One can speculate that the unique atrial specific distribution of α_1D_ Ca channel, together with the documented inhibitory effect of the anti-Ro antibodies on the α_1D_ Ca channels, may suggest that anti-Ro positive patients might be at increased risk of having atrial fibrillation, warranting further investigations.

## Conclusions and Future Directions

Cardiac Ca channels, including both L- and T-type Ca channels, play critical roles in the impulse generation in the SAN, the conduction through the AVN and the development of arrhythmias. Autoantibodies targeting Ca channels have been identified in 2 major pathologies, DCM and CHB. In addition, several autoantibodies are directly related to sudden death in patients with DCM, including anti-N/K-ATPase, anti-M2 muscarinic acetylcholine receptors, and anti-β1 receptor antibodies, indirectly affecting the L-type VGCCs. Early risk stratification to effectively prevent adverse outcomes in DCM has been challenging. Recent studies confirmed the presence of autoantibodies directly against Ca channel α_1C_ subunit in DCM, which was identified as a strong predictor for ventricular arrhythmias and sudden cardiac death, indicating that anti-α_1C_ Ca channel antibodies might be a valuable biomarker to predict sudden death in DCM.

**Figure 3:**
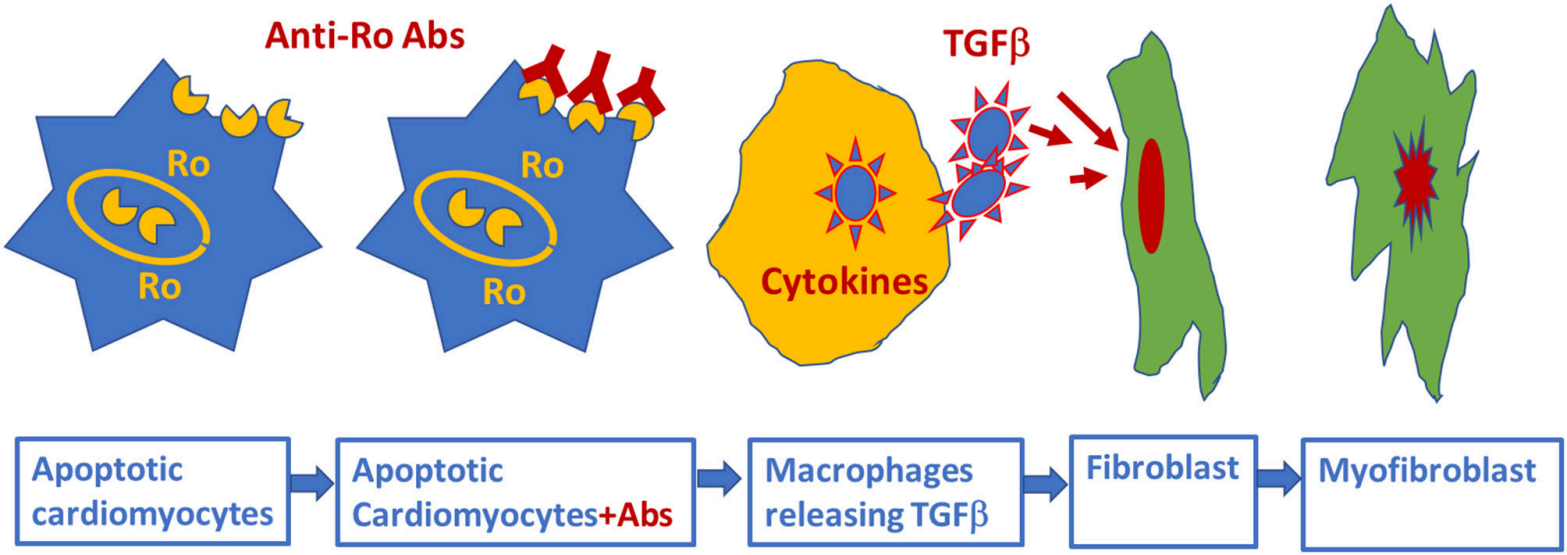
Schematic representation of alternative mechanism of linking anti-Ro antibodies to the development of atrioventricular block: fetal cardiomyocytes undergoing “physiological” apoptosis cause the surface translocation of the intracellular located Ro antigens. Circulating maternal anti-Ro antibodies which can cross the placenta, subsequently bind to the translocated Ro antigens at the cell surface; provoke the secretion of proinflammatory cytokines such as TGFβ from macrophages. Excessive TGFβ secretion activates fibroblasts leading to scars promoting myofibroblasts in the Atrioventricular node, resulting in atrioventricular block.

**Figure 4:**
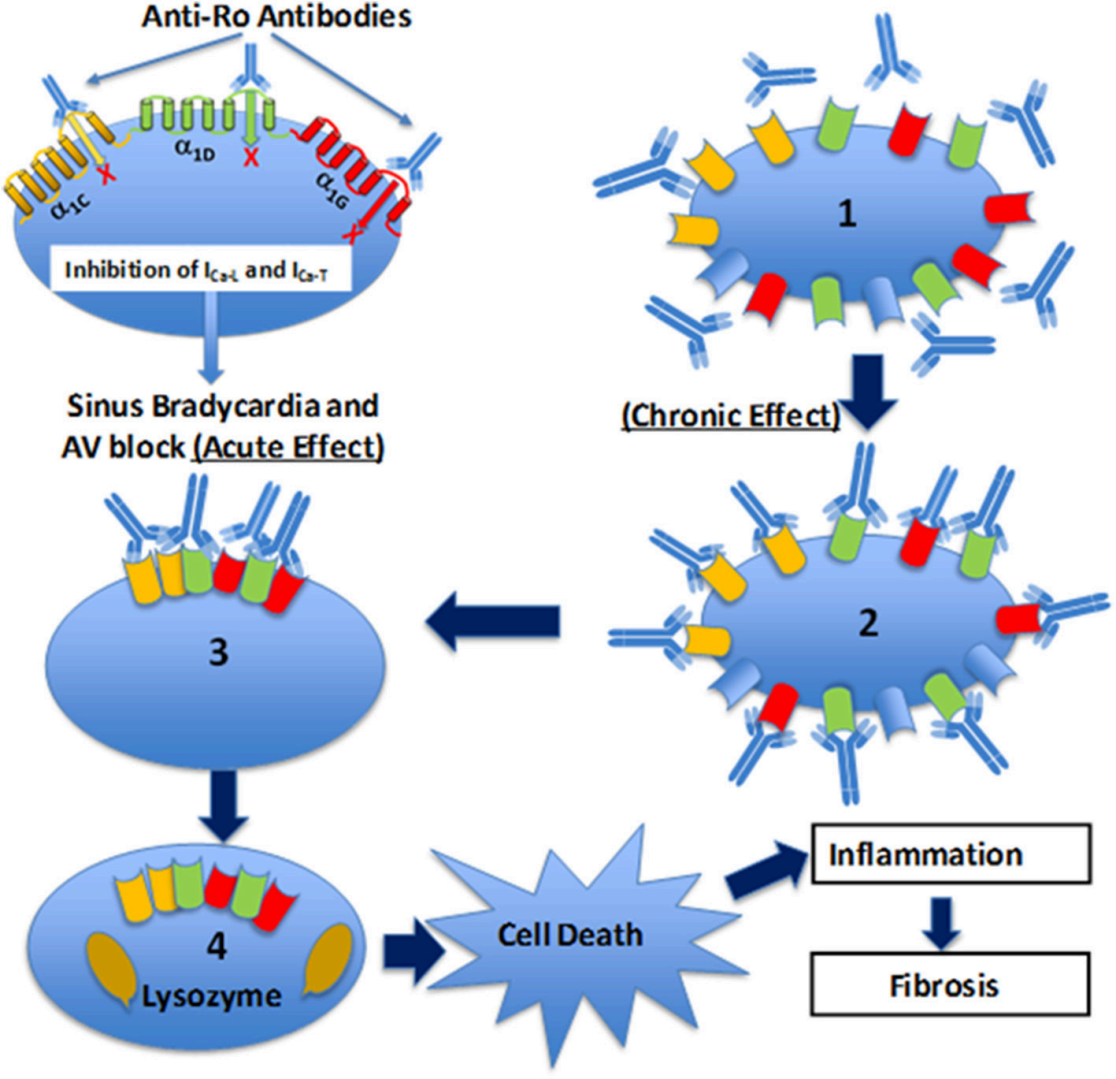
Schematic illustration of the Ca channel hypothesis. Maternal anti-Ro antibodies cross react and bind to α_1C_ (yellow), α_1D_ (green), and α_1G_ (red) Ca channels in the fetal human heart, inhibit all three Ca currents leading to sinus bradycardia and atrioventricular (AV) block (acute effect). Furthermore, fetal heart Ca channels are exposed *chronically* (chronic effect) (1) to maternal anti-Ro antibodies during pregnancy. Binding of anti-Ro antibodies to Ca channels (2), can cause cross-linking of the adjacent ion channels by the two Fab arms of IgG (3) to increase the internalization of the channel/antibody complex and thereby decrease of the channel density on the cell membrane. Internalized Ca channels are lysed by lysosomes (4). If the number of Ca channels on cell surface decreased to a critical level, then cell death will occur. Cell death, *per se*, could trigger inflammation subsequent to leukocytic influx resulting in damage of the surrounding healthy myocytes such as in sinoatrial node and AV node which can cause permanent sinus bradycardia and AV block.

The association of anti-Ro autoantibodies with CHB is generally accepted, but the predictive value of these autoantibodies is still low despite overwhelming experimental data demonstrating causality between anti-Ro antibodies and electrocardiographic abnormalities seen in CHB (Figure 2). This indicates that anti-Ro antibodies are necessary, but not sufficient, for inducing the clinical electrocardiographic phenotype. To date, two hypotheses have been proposed to explain the molecular mechanism(s) by which maternal anti-Ro antibodies lead to the development of CHB in the fetal hearts (79, 121). The “apoptosis hypothesis” (Figure 3) suggests that intracellular antigens translocate to the surface of cardiomyocytes undergoing apoptosis during physiological remodeling, thereby exposing the antigens to the circulating maternal anti-Ro antibodies. Binding of anti-Ro antibodies to the cell surface antigens promotes pro-inflammatory and pro-fibrotic responses (122, 123), causing the fibrosis of the AVN, which eventually leads to the development of the irreversible AVB (124, 125). The “Ca channel hypothesis” explained in this review is based on molecular mimicry, whereby anti-Ro antibodies directly cross-react and subsequently inhibit the cardiac Ca channels' activity, thereby causing sinus bradycardia and AVB (77, 78, 108) (Figure 4). This occurs by anti-Ro autoantibodies binding to Ca channels and the resulting inhibition of I_CaL_ (Acute effect, Figure 4). The subsequent cross-linkage and downregulation of Ca channels and lysis by lysosomes followed by intracellular Ca dysregulation leads to cell death/apoptosis, inflammation, and fibrosis of the AVN (Figure 4). The ultimate proof of direct autoantibodies' involvement in CHB is provided by the identification of the site of action on the different subunits of cardiac Ca channels (126–128), including α_1C_ and α_1D_ subunits of L-type VGCCs and α_1G_ subunit of T-type VGCCs (Figure 4). Although autoantibodies are utilized as diagnostic or prognostic markers in other pathologies, unfortunately, to date, there is no specific maternal marker for assessing the increased risk of having CHB children during an anti-Ro positive pregnancy. It is possible that, instead of having a single CHB-inducing antibody specificity, future studies may focus on several different specificities that may act synergistically to induce AVB in fetal hearts.

Peptide-based therapeutic approaches are one of the growing classes of novel therapeutic agents. The development of short non-immunogenic peptides and their use as decoy targets for pathogenic autoantibodies is expected to minimize and/or prevent autoantibody association with ion channels and their functions. This therapeutic path awaits further development and progress.

## Author Contributions

All authors listed have made a substantial, direct and intellectual contribution to the work, and approved it for publication.

### Conflict of Interest Statement

The authors declare that the research was conducted in the absence of any commercial or financial relationships that could be construed as a potential conflict of interest.
